# Longitudinal assessment of inflammatory markers in the peripartum period by depressive symptom trajectory groups

**DOI:** 10.1016/j.bbih.2022.100468

**Published:** 2022-05-02

**Authors:** Emma Bränn, Alkistis Skalkidou, Jaclyn Schwarz, Fotios C. Papadopoulos, Inger Sundström Poromaa, Emma Fransson

**Affiliations:** a Department of Women's and Children's Health, Uppsala University, Uppsala, Sweden; b Department of Psychological and Brain Sciences, University of Delaware, USA; c Department of Neuroscience, Psychiatry, Uppsala University, Uppsala, Sweden; d Centre for Translational Microbiome Research, Department of Microbiology, Tumor and Cell Biology, Karolinska Institutet, Stockholm, Sweden

**Keywords:** Pregnancy, Immune response, Depression, Psychoneuroimmunology, IL, Interleukin, TNF, Tumor necrosis factor, M-CSF, Macrophage colony-stimulating factor, VEGF-A, Vascular endothelial growth factor A, EPDS, Edinburgh Postnatal Depression Scale, APD, Antepartum depressive symptoms, PPD, Postpartum depressive symptoms

## Abstract

**Objective:**

Mechanisms driving temporal fluctuations of inflammatory markers during pregnancy, and how these might differ between distinct perinatal depressive trajectories, are not well understood. The aim of this study was to investigate cytokines levels over the course of pregnancy in women with different trajectories of depressive symptoms peripartum, and relate the levels to levels of non-pregnant controls.

**Methods:**

Based on the Edinburgh Postnatal Depression Scale and/or selective serotonin reuptake inhibitors use, 131 women were categorized into: no (n = 65); antepartum (APD, n = 19), postpartum (PPD, n = 17) and persistent (n = 30) depressive symptoms. Plasma samples (n = 386) were analyzed for levels of interleukin (IL)-8, IL-18, Tumor necrosis factor-α, macrophage colony-stimulating factor (M-CSF), vascular endothelial growth factor A (VEGF-A) and fractalkine, at four different time-points (twice during pregnancy, during childbirth, and postpartum) using Bio-Plex Pro Human Cytokine Assays. Generalized linear mixed models were applied to analyze the associations between cytokine levels, time-point, perinatal depressive symptom trajectory group and their interaction.

**Results:**

For all markers but VEGF-A, pregnancy was associated with higher cytokine levels compared to the non-pregnant controls, with delivery being the most prominent time-point. For M-CSF, IL-18 and VEGF-A, levels were back to the non-pregnant status at postpartum week 8. An effect of perinatal depressive symptom trajectory groups on cytokine levels was found for VEGF-A. Women with PPD and women with APD had lower levels of VEGF-A throughout the study period compared to women with persistent depression, and women with PPD had lower levels compared to non-depressed women.

**Conclusions:**

Lower levels of VEGF-A were noted among women in some trajectories of depressive symptoms peripartum. The peripartum period is a time of tremendous immune system adaptations. Standardization of time-points for cytokine measurements in studies of perinatal depression are important in order to draw valid conclusions on the role of the immune system in perinatal depression.

## Highlights

• Sampling time-points for cytokine assessment are important in perinatal populations.
• VEGF-A levels vary in different perinatal depressive symptom trajectory groups.
• Most cytokines reach non-pregnant control levels at eight weeks postpartum.

## 1 Introduction

Perinatal depression, defined by the Diagnostic and Statistical Manual of Mental Disorders 5 (DSM-5, 2013) as a major depressive disorder with peripartum onset, affects about 10% of women giving birth (Woody et al., 2017). Perinatal depression comes with a high societal cost (Bauer et al., 2016) with substantial consequences for the mother (Slomian et al., 2019), the partner (Cameron et al., 2016; Paulson and Bazemore, 2010) and the child (Agnafors et al., 2013a, 2013b; Fransson et al., 2020). Fluctuations in steroid hormonal levels (Bloch et al., 2003), in hypothalamic–pituitary–adrenal-axis regulation of glucocorticoids (Glynn et al., 2013; Hannerfors et al., 2015; Iliadis et al., 2015; Jolley et al., 2007) and alterations in oxytocin levels (Skrundz et al., 2011; Stuebe et al., 2013) are some of the factors studied for identification of the underlying mechanism of perinatal depression. Other areas of interest include neurological plasticity (reviewed in (Brummelte and Galea, 2016) and altered neurotransmitter levels (Roomruangwong et al., 2018). A similarity amongst all of these factors are that they rely on the ability to adapt to the major physiological and psychological changes that the pregnancy and postpartum period entails.

One of the greatest changes during pregnancy is the adaptation of the immune system (Gillespie et al., 2016; Hedman et al., 2020; Mor et al., 2011; Racicot et al., 2014; Sherer et al., 2017). The immune system takes on the task of continuing the search and elimination of pathogens, while at the same time not rejecting the semi-allogenic fetus. While this task was previously considered immune suppressive, later data suggests that the immune system needs to be active and responsive to promote a successful pregnancy (Mor et al., 2017). Pregnancy is characterized by different immunological stages, starting with a pro-inflammatory state that later converts to an anti- or regulatory inflammatory state and ends up going back to a pro-inflammatory state prior to the delivery (Mor and Cardenas, 2010; Osborne et al., 2019). The postpartum period could also be divided into three phases: the first hours after birth when rapid changes occur; the first 2–6 weeks when the body is undergoing major changes and recovering; and the late phase that could extend into the first half year after birth (Romano et al., 2010). Our research group has previously shown the tremendous change of the immune system from pregnancy to postpartum with drastic change in peripheral immune markers (Bränn et al., 2019). The key to a healthy pregnancy seems to lie in the adaptation of different body systems to the different stages of pregnancy, as well as the body's ability to recover from these adaptations after delivery. Excess inflammation has been linked to poor pregnancy outcomes such as preeclampsia (Ahn et al., 2011), preterm birth (Wei et al., 2010), and gestational diabetes (Bo et al., 2005; Wolf et al., 2004). As elevated inflammatory markers now have an established association with depression outside the perinatal period (Enache et al., 2019; Iob et al., 2020; Osimo et al., 2020; Patel, 2013), the role of inflammation and immune system adaptations in the etiology of perinatal depression is not to be under-estimated (Leff-Gelman et al., 2016).

Previous results have pointed to specific attributes of immune activation in depression during pregnancy, such as *lower* regulatory markers of inflammation in women with antepartum depression (Edvinsson et al., 2017). Further, postpartum depression is potentially proceeded by *lower* levels of anti-inflammatory or regulatory markers in late pregnancy (Bränn et al., 2017), while an *elevated* pro-inflammatory profile seems associated with postpartum depressive symptoms (Bränn et al., 2018). Previous studies focusing on depressive symptoms in the peripartum period in humans most often focus on cross-sectional measures at one time point (Achtyes et al., 2020; Blackmore et al., 2011; Bränn et al., 2017, 2018; Christian et al., 2010; Edvinsson et al., 2017; Maes et al., 2000; Shelton et al., 2015; Skalkidou et al., 2009). While each of these studies contributes to the understanding of the complex puzzle of perinatal depression, the results are inconclusive, perhaps suggesting that individual levels of inflammatory markers may be less important than the overall immune adaptation.

A few previous studies have reported results across two or more time points: Osborne et al. (2019) investigated 23 cytokines related to innate immunity across pregnancy and postpartum and found interleukin (IL)-6, IL-15 and CCL3 to be higher in more severely depressed women in the third trimester. Simpson et al. (2016) found no differences in IL-6, IL-10 and Tumor necrosis factor (TNF)-α across time in women with and without depressive symptoms. Further, Corwin et al. (2015) studied the interaction of the hypothalamic–pituitary–adrenal axis and levels of cytokines, measured once during pregnancy and six times postpartum, and found the area under curve for cortisol levels, and IL8/IL10 ratio at two weeks postpartum to be predictors of postpartum depression at six months postpartum. Krause et al. (2014) found regulatory T cells to be increased during both pregnancy and postpartum in women with postpartum depressive symptoms. Animal models of peripartum depression have shown that elevated levels of IL-6 in the brain are associated with increased anhedonia immediately postpartum (Gomez et al., 2019) and persistent postpartum anhedonia has been shown to be precipitated by stress during gestation (Haim et al., 2017). More recently, peripartum depression has been characterized as having potentially different sub-types, depending partly on time of onset and persistence of symptoms (Jacques et al., 2020; Mora et al., 2009; Putnam et al., 2017; Wikman et al., 2020). These studies suggest the sub-types, defined as antepartum depressive symptoms, postpartum depressive symptoms, or persistent depressive symptoms, have different trajectories as well as different sets of risk factors. Risk factors relating to background characteristics (such as age, education, and employment) were more strongly associated with depressive episodes during pregnancy and persistent depression. Delivery related risk factors (such as delivery experience rated as negative, and instrumental delivery) were associated with postpartum depressive symptoms with onset at six weeks postpartum. Postpartum risk factors (such as sleep loss, lack of support from partner, and maternal-infant bonding difficulties) were associated with both postpartum depressive symptoms with onset at six weeks postpartum and with onset at 6 months postpartum, as well as persistent depression. The differences in risk factors within the different perinatal depressive symptom trajectories suggest distinct pathophysiology; a concept not previously considered in the studies of immunological alterations across the perinatal period mentioned above.

With regard to knowledge of the perinatal depressive symptom trajectories, the association between depressive symptoms and the inflammatory response adaptation across various points of pregnancy, as well as the relation to non-pregnant controls, gaps are present. We hypothesize that levels of inflammatory markers differ across peripartum time points, and furthermore, that levels of inflammatory markers in women with different depressive symptom trajectories (antepartum depressive symptoms, postpartum depressive symptoms or persistent depressive symptoms) differ from non-depressed pregnant women, and from non-depressed non-pregnant women.

The aim of this study was to investigate if non-depressed pregnant women and women with different trajectories of depressive symptoms show different patterns of change in peripheral markers of inflammation across pregnancy and postpartum and to further to illustrate these changes by graphically presenting them against healthy non-pregnant controls.

## 2 Methods

### 2.1 Participants and sample collection

#### 2.1.1 Sample collection

Data from pregnant and postpartum women were drawn from the BASIC study (Biology, Affect, Stress, Imaging and Cognition) a population-based longitudinal cohort study that recruited pregnant women from Uppsala, Sweden from 2009 to 2018 (Axfors et al., 2019). All Swedish speaking women ≥18 years of age who were scheduled for a routine ultrasound at Uppsala University Hospital were invited to participate. In the BASIC study, web-based surveys including questions on background characteristics, medication use, and self-reported mental health were filled out around gestational weeks 17 and 32, as well as 6 weeks postpartum. Further, participants were asked if they were willing to donate biological samples at time of delivery.

Moreover, at gestational week 38 and in postpartum week 8, a subgroup of women in the BASIC study (either with or without clear depressive symptoms) were invited to participate in a sub-study which included neuropsychiatric interviews, self-reported mental health screening, and additional blood sample collection through routine venipuncture (Axfors et al., 2019). Furthermore, women in the BASIC study scheduled for pre-labor cesarean section at Uppsala University Hospital were asked to donate a blood sample prior to surgery (in this study grouped together with the gestational week 38 samples, n = 15). In addition, some of the women participating in the BASIC study had also donated blood to the Uppsala Biobank for Pregnant Women at approximately gestational week 18.

In summary, the chronological order of possible blood donation throughout the perinatal period within the BASIC-study was as follows: **1**. at gestational week 18 via the Uppsala Biobank for Pregnant Women, **2.** at gestational week 38/prior to cesarean section, **3.** in conjunction with labor and **4.** at postpartum week 8. Notably, not all women were sampled at all time-points. Samples were kept at room temperature for a maximum of 1 h before being centrifuged for 10 min in 1500 RCF (relative centrifugal force). The plasma was separated and stored at −70 °C until the time of cytokine assessment, performed for all samples simultaneously.

#### 2.1.2 Perinatal depressive symptom trajectory groups

For this study, data on depressive symptoms were detected using the Edinburgh Postnatal Depression Scale (EPDS) (Cox et al., 1987) filled in by the women in the BASIC study at gestational weeks 17, 32 and 38, as well as at 6 and 8 weeks postpartum. The EPDS has a specificity of 88.0% and a sensitivity of 72.0% in the Swedish context (Swedish Agency for Health Technology Assessment and Assessment of Social Services SBU, 2012) and is widely used in both research and healthcare.

From the EPDS scores filled out in at gestational weeks 17, 32 and 38, and at 6 and 8 weeks postpartum, the women included in this study were categorized into four different perinatal depression trajectory groups. Notably, the assessment with EPDS was not performed at the exact same timepoint as the blood draw. In addition, based on previous findings from our research group, where no differences in immunological markers between women with depressive symptoms and women with selective serotonin reuptake inhibitors (SSRIs) were observed (Edvinsson et al., 2017), five women with anti-depressive medication at time of blood sampling were included in depressive groups based on a combination of high EPDS scores and use of antidepressants, or solely on the use of antidepressants.

Women were categorized as no depressive symptoms (**non-depressed**; EPDS<12 at all pregnancy and postpartum surveys and no SSRI treatment, n = 65), antepartum depressive symptoms (**APD**; EPDS≥12 at gestational weeks 17, 32 or 38, or SSRI treatment during pregnancy but not postpartum, n = 19 whereof one woman was included based solely on antidepressants use during pregnancy), postpartum depressive symptoms (**PPD**; EPDS≥12 at 6 or 8 weeks postpartum, or SSRI treatment postpartum but not during pregnancy, n = 17) and persistent depressive symptoms (**persistent**; EPDS≥12 at gestational weeks 17, 32 or 38 and at 6 or 8 weeks postpartum, or SSRI treatment at both pregnancy and postpartum, n = 30 whereof two women were included based on a combination of high EPDS scores and antidepressant use (high EPDS scores in pregnancy – antidepressants postpartum, and antidepressants during pregnancy – high EPDS scores postpartum), and two based on antidepressant use alone.

For the purpose of this sub-study, samples from women who donated two or more blood samples throughout pregnancy, delivery or postpartum and who filled out the EPDS at least once during pregnancy and once postpartum were analyzed. Furthermore, women who reported taking antibiotics and glucocorticoids at the time of blood sampling were excluded from the analyses as both these drugs are administrated for suppression of inflammation, leaving an analytic sample of 131 perinatal women and 386 plasma samples.

#### 2.1.3 Healthy controls

In addition, healthy non-pregnant controls (n = 53) aged 22–38 years old, with body mass index (BMI) within the range of 20–29 kg/m^2^, with parity less than 4, and with no systemic disease or current psychiatric disease were invited to the BASIC study in form of a thorough assessment similar to the BASIC protocol, at the Women's and Children's Research Clinic. Additional inclusion criteria were that the women had never, or not during the past two years, given birth and had completed breastfeeding at least 3 months prior to participation. Further, the healthy controls reported having regular periods, to not be using any contraceptives or to be using combined contraceptives or IUD, and to not be suffering from premenstrual syndrome. To obtain a homogenous sample, the non-pregnant healthy controls were invited for the assessment in the luteal phase (day 16–26) of their menstrual cycles. At the assessment, a survey including questions of background characteristics and the EPDS were filled in, and venous blood samples were collected.

### 2.2 Confounding factors

Confounding factors were collected from the survey sent out at gestational week 17, and from medical records. Age at time of delivery, pre-pregnancy BMI, educational level (university or lower), employment status (employed/studying/parental leave or sick leave/unemployed), parity (nulliparous or not) and a self-reported history of a depressive episode (yes or no) were investigated as possible confounders by directed acyclic graph (DAG) analysis (http://www.dagitty.net/).

### 2.3 Ethical considerations

This study was approved by the Ethical Review Board in Uppsala (Dnr, 2010/171 with amendments for the BASIC-study and Dnr, 2007/181 for Uppsala Biobank for Pregnant Women). All participants gave their written, informed consent to the Uppsala Biobank for Pregnant Women, to the BASIC study, and to the sub-study prior to inclusion.

### 2.4 Analyses of inflammatory markers

Plasma samples were analyzed for levels of ten cytokines; (interleukin (IL)-1beta, IL-4, IL-6, IL-8, IL-10, IL-18, TNF-α, macrophage colony-stimulating factor (M-CSF also named CSF-1), vascular endothelial growth factor A (VEGF-A) and fractalkine (or chemokine (C-X3-C motif) ligand 1; CX3CL1) using Bio-Plex Pro Human Cytokine Assays – 9-plex (Cytokine Screening Panel 1) and a single-plex for fractalkine (https://www.bio-rad.com/bio-plex). The method is similar to that of a sandwich ELISA with antibodies coupled to magnetic beads, and further a secondary detection antibody is added. Analyses were performed at SciLifeLab, Science for Life Laboratory, Solna, Sweden. Values below LOD, were replaced by the LOD value divided by the square root of two (National Center for Health Statistics, 2013). Four out of 10 markers (IL-1beta, IL-4, IL-6 and IL-10) had levels under the lower limit of detection (LLOD) for more than 25% of the samples and were excluded from the statistical analyses. For the remaining cytokines, 6.4% were imputed for IL-8, 0.2% for TNF-α, and 22.1% for VEGF-A. For IL-18, M-CSF and fractalkine no imputation was performed.

### 2.5 Statistical analyses

Univariate analyses for background characteristics were performed using Chi-square test for categorical variables and ANOVA and Kruskal-Wallis test for continues variables as suited. DAG analyses suggested age, pre-pregnancy BMI, and education to be included as confounding factors.

Median cytokine levels over time for the different perinatal depressive symptom trajectory groups were plotted in SPSS version 27. A references line representing median levels of the cytokine for non-pregnant controls was added. Mann-Whitney U tests were applied to compare pregnant samples to non-pregnant controls. To explore the data, Spearman's correlations analyses were applied for assessment of correlations between time-points and different cytokines. Histograms and analyses for skewness and kurtosis were applied to evaluate normal/skewed distributions.

Generalized linear mixed models fitted with gamma distribution and log link function were applied. This method was chosen as not all women had been sampled at all time-points, as there was correlation of levels of markers between the different time-points, and as data presented non-linearity and non-normal distribution. Main effect coefficients for time-point, perinatal depressive symptom trajectory group and an interaction term between time-point and perinatal depressive symptom trajectory group were computed. Furthermore, the covariates: age at time of delivery, pre-pregnancy BMI, and educational level were included in the model as fixed effects, while the intercept of each woman was set as random. Because of the complexity of the model, and a relatively small sample size per group and time-point, we followed up main effects and interaction with p-values <0.1 by use of the pairwise post-hoc tests provided in the generalized linear mixed models. All analyses were performed using SPSS versions 27.

Furthermore, two additional sensitivity analyses were conducted in accordance with the main analysis where (1) the five women grouped solely on the use of antidepressants were excluded, and (2) the delivery time-point was excluded.

## 3 Results

The 131 included women were categorized as a) having no depressive symptoms (n = 65), b) only antepartum depressive symptoms, APD (n = 19), c) only postpartum depressive symptoms, PPD (n = 17), and d) Persistent depressive symptoms (n = 30). A group of healthy non-pregnant women with no history of or current depression (n = 53) were included in the graphical visualization. Background characteristics and number of analyzed blood samples per time-point are presented in Table 1.

**Table 1:** Descriptive data of background characteristics.

	No depressive symptoms	Antepartum depressive symptoms	Postpartum depressive symptoms	Persistent depressive symptoms	Non pregnant controls
n women =	65	19	17	30	53
n samples =	197	53	47	89	53
Age, mean (SD)	31.6 (4.4)	31.2 (4.3)	33.8 (3.4)	30.9 (5.3)	29.5 (5.7)
BMI, median (IQR)	22.5 (2.8)	22.5 (3.5)	23.4 (6.5)	23.7 (5.7)	23.1 (3.9)
Employment sick leave or unemployed, n (%)	0 (0.0)	1 (6.3)	1 (5.9)	3 (10.7)	0 (0.0)
Education lower than university, n (%)	7 (11.7)	5 (31.3)	2 (11.8)	8 (28.6)	9 (17.0)
Nulliparous, n (%)	37 (56.9)	9 (47.4)	7 (41.2)	20 (66.7)	28 (52.8)
History of depression	21 (33.9)	13 (76.5)	12(70.6)	25 (89.3)	13 (24.5)
SSRI n (%)	0 (0.0)	2 (10.5)	2 (11.8)	14 (46.7)	0 (0.0)
Samples gestational week 18, n	52	14	14	17	0
Samples gestational week 38, n	23	17	7	20	0
Samples delivery, n	59	15	12	23	0
Samples postpartum, n	63	7	14	29	0
Samples non pregnant control, n	0	0	0	0	53

SD = standard deviation, BMI = body mass index, IQR = inter quartile range, SSRI = selective serotonin reuptake inhibitor.

**Fig. 1:**
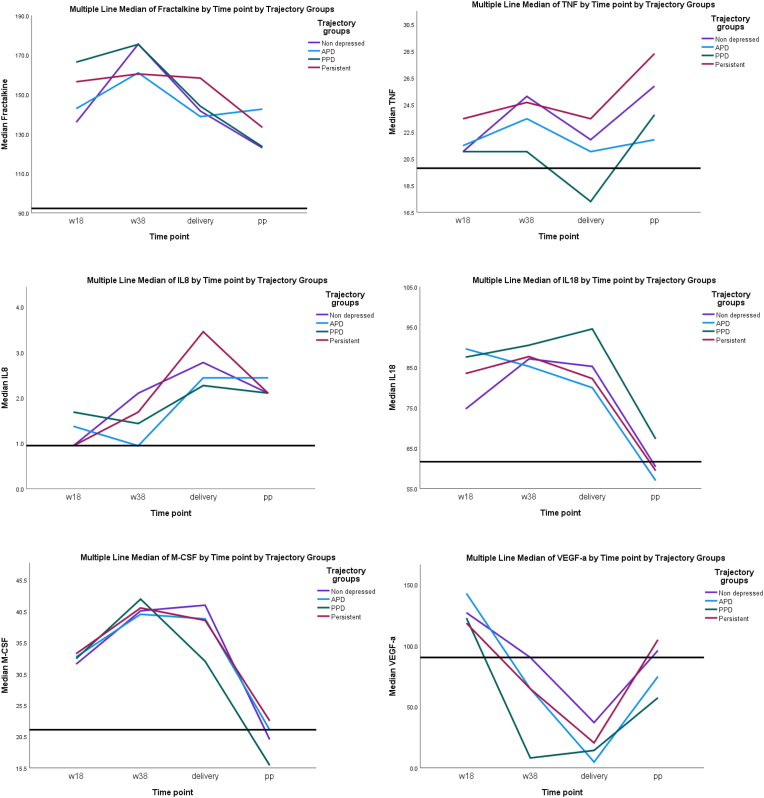
Median cytokine levels over time for the four trajectory groups compared with a reference line representing the median levels of healthy non-pregnant controls.

Median cytokine levels across the perinatal period for the four perinatal depressive symptom trajectory groups compared with a reference line representing the median levels of healthy non-pregnant controls are presented in Fig. 1. IL-8 displayed a drastic increase at the time of delivery across all groups (p < 0.001). M-CSF increased already during late pregnancy with continuous high levels through delivery (p < 0.001), while fractalkine and TNF-α increased in late pregnancy (p < 0.001) and dropped at delivery, but did not reach levels of non-pregnant women postpartum (p < 0.001). For all markers but VEGF-A, pregnancy led to an increase in cytokine levels compared to the non-pregnant controls and for M-CSF, IL-18 and VEGF-A the levels were back to non-pregnant levels at postpartum week 8 (p ≥ 0.089).

Spearman correlation analyses showed that most of the different cytokines were positively correlated. Further analyses showed significant correlations between most of the cytokines at all time points (Supplementary Table 1).

Crude as well as adjusted analysis of cytokine levels in the four perinatal depressive symptom trajectory groups across the perinatal period by generalized linear mixed models, showed significant main effects of time-point for all cytokines; Fractalkine F(3,336) = 5.10; p < 0.01, TNF-α F(3,338) = 3.27; p < 0.01, IL-8 F(3,338) = 14.26; p < 0.001, IL-18 F(3,338) = 13.55; p < 0.001, M-CSF F(3,338) = 73.52; p < 0.001, and VEGF-A F(3,338) = 18.38; p < 0.001 (Table 2). For most of the cytokines, levels differed significantly between the majority of the time-points (Supplementary Table 2).

**Table 2:** Coefficients (Main effects) from generalized linear mixed models including time point and trajectory group as well as their interaction, unadjusted and adjusted for potential confounders.

	Fractalkine								TNF-α							
	Fixed Effects				Fixed Effects^a^				Fixed Effects				Fixed Effects^a^			
	F	df1	df2	Sig.	F	df1	df2	Sig.	F	df1	df2	Sig.	F	df1	df2	Sig.
Trajectory group	0.162	3	368	0.922	0.320	3	336	0.811	1.258	3	370	0.289	1.853	3	338	0.137
Time point	5.507	3	368	<**0.001**	5.098	3	336	**0.002**	5.046	3	370	**0.002**	3.273	3	338	**0.021**
Interaction Trajectory group * Time point	0.556	9	368	0.833	0.568	9	336	0.823	1.262	9	370	0.256	1.284	9	338	0.244
	**IL-8**								**IL-18**							
	Fixed Effects				Fixed Effects^a^				Fixed Effects				Fixed Effects^a^			
	F	df1	df2	Sig.	F	df1	df2	Sig.	F	df1	df2	Sig.	F	df1	df2	Sig.
Trajectory group	1.380	3	370	0.249	1.245	3	338	0.293	0.285	3	370	0.836	0.030	3	338	0.993
Time point	13.984	3	370	**<0.001**	14.257	3	338	**<0.001**	13.233	3	370	**<0.001**	13.547	3	338	**<0.001**
Interaction Trajectory group * Time point	1.319	9	370	0.225	1.370	9	338	0.200	0.780	9	370	0.635	0.813	9	338	0.604
	**M-CSF**								**VEGF-A**							
	Fixed Effects				Fixed Effects^a^				Fixed Effects				Fixed Effects^a^			
	F	df1	df2	Sig.	F	df1	df2	Sig.	F	df1	df2	Sig.	F	df1	df2	Sig.
Trajectory group	1.452	3	370	0.227	1.966	3	338	0.119	2.283	3	370	**0.079**	2.311	3	338	**0.076**
Time point	76.738	3	370	**<0.001**	73.511	3	338	**<0.001**	18.024	3	370	**<0.001**	18.376	3	338	**<0.001**
Interaction Trajectory group * Time point	1.396	9	370	0.188	1.606	9	338	0.112	0.953	9	370	0.479	0.991	9	338	0.447

Probability distribution: Gamma.

a Adjusted for age, BMI and educational level.

Main effects of perinatal depressive symptom trajectory groups on cytokine levels were found for VEGF-A (F(3,370) = 2.28; p < 0.1) in the unadjusted model. The main group effects for levels of VEGF-A remained significant after adjustments for age, BMI and educational level (F(3,338) = 2.31; p < 0.1). Pairwise post-hoc tests showed VEGF-A levels to be significantly lower throughout the study period for both APD and PPD compared to persistent. Further, women with PPD had significantly lower VEGF-A throughout the study course compared with non-depressed women (Table 3 and Fig. 1).

**Table 3:** Pair-wise post-hoc tests for VEGF-A and trajectory groups, adjusted for age, BMI and educational level. P-values <0.01 marker in bold.

Pairwise Contrasts VEGF-A							
Trajectory Groups Pairwise Contrasts	Contrast Estimate	Std. Error	t	df	Adj. Sig.	95% Confidence Interval	
						Lower	Upper
Persistent - PPD	0.459	0.220	2.087	338	**0.038**	0.026	0.892
Persistent - APD	0.425	0.216	1.971	338	**0.050**	0.001	0.849
Persistent - Non depressed	0.115	0.164	0.701	338	0.484	−0.207	0.436
PPD - APD	−0.034	0.248	−0.137	338	0.891	−0.523	0.455
PPD - Non depressed	−0.344	0.202	−1.705	338	**0.089**	−0.742	0.053
APD - Non depressed	−0.310	0.198	−1.565	338	0.118	−0.701	0.080

The least significant difference adjusted significance level is 0.05.

No significant interactions between perinatal depressive symptom trajectory groups and time point on cytokine levels were found in the overall analyses (Table 2), suggesting that fluctuation of cytokine levels was unaffected by depression trajectory.

For sensitivity analysis 1, where the five women who were grouped based on the use of antidepressant medication solely were excluded, the results remained the same except for VEGF-A, where the main effects of perinatal depressive symptom trajectory groups were no longer significant (F(3,324) = 2.02; p = 0.111).

In sensitivity analysis 2, where samples from the delivery time point were excluded, the results remained the same.

## 4 Discussion

This study investigated levels of inflammatory markers throughout pregnancy and postpartum by different perinatal depressive symptom trajectories. As expected, major differences in levels of all cytokines across the perinatal time period were found. We also noted a group difference in levels of VEGF-A, with lower levels throughout the study period in women with APD and PPD compared to persistent depression, and lower levels in women with PPD compared to non-depressed. However, we detected no evidence that changes in cytokine levels were associated with any of the perinatal depressive symptom trajectory groups.

The first major finding regards the dramatic changes in inflammatory markers across the study period for the investigated immune-related markers that highlights the importance of timing of blood sampling in studies of inflammatory markers in a perinatal population. The results present immune changes over the perinatal time period with the delivery representing an expected pro-inflammatory event, and the 8-week postpartum time-point a return of most markers back to the non-pregnant state. This fluctuation of cytokines across the perinatal period remained significant even after excluding samples collected at the delivery time point. Among our findings, at the 8-week postpartum time-point, most markers had decreased to nearly non-pregnant levels, in contrast with a previous study that reported a continuous increase of IL-6 from pregnancy into the post-partum period (Christian and Porter, 2014). However, we collected our samples at eight weeks postpartum, while the previous study sampled women between weeks 4–6 postpartum. In the present study, we also added visual comparisons with non-pregnant women and four of the markers displayed “normal” median levels (not different from non-pregnant females) by the 8-week postpartum sampling time point. The intricate changes of the immune system during pregnancy has been described with accumulating details and precision, i.e. by Gil Mor et al. (2011), while the postpartum period is less well characterized. In a previous study, we show indications of a drop in inflammatory markers at a similar time point as in the present study (Bränn et al., 2019). We could speculate that the pro-inflammatory event of the delivery, followed by wound healing of damaged tissue might be largely resolved by this point. As with pregnancy, the postpartum period has been suggested to have three phases, one acute, one intermediate that ends by approximately six weeks, and then a prolonged phase that might be ongoing for the first half year (Romano et al., 2010).

The second major finding regards differences in the temporal cytokine measures between depression trajectory groups. Despite differences in median levels between groups, the only group effects that were seen in the statistical modeling were for VEGF-A. During pregnancy, VEGF-A is believed to be involved in angiogenesis and remodeling of the uterus and to be critical in placental formation and development (Chen and Zheng, 2014). Women with APD and PPD presented lower levels of VEGF-A throughout the perinatal period compared to levels of women with persistent depressive symptoms. Further, women with PPD presented lower levels of VEGF-A throughout the perinatal period compared to non-depressed. While this result no longer remained significant when the five women who were grouped based on the use of antidepressant medication solely were excluded, the direction of the estimate were the same, suggesting a power issue. The result is also in line with a previous study from our group, where lower pregnancy levels of VEGF-A have been associated to antenatal depression (Edvinsson et al., 2017). Outside of pregnancy, a meta-analysis showed that VEGF-A could be used as a marker for major depression as it is found to be *elevated* in depressed groups (Carvalho et al., 2015). The persistently depressed women have previously been characterized as slightly different from antenatal and postpartum depressed women (Jacques et al., 2020; Mora et al., 2009; Wikman et al., 2020) suggesting a different pathology, which could be unrelated to immunological adaptation. The sub-grouping of perinatal depression trajectory groups in our study could potentially explain the differences in results found in the different studies. Dissimilar measures of depressive symptoms and techniques used, as well as slightly different time points could further explain the contradictory results. However, more in line with our findings, a recent study showed that antidepressant effect of ketamine might be mediated by increase in VEGF as well as in brain-derived neurotrophic factor (BDNF) (Deyama et al., 2019). Notably, VEGF-A is expressed in the placenta throughout pregnancy, and blockage of VEGF-A signaling pathways has shown to reduce regulatory T cells (Terme et al., 2013); cells involved in the maintenance of pregnancy (Zenclussen, 2006).

No general interaction effects between perinatal depressive symptom trajectory group and time point were observed. This negative finding might be due to the limited samples size, as well as the fact that there is large interindividual variation within each trajectory group and time-point.

It has previously been found that the inflammatory phenotype of depression might constitute a big proportion, but still not all, of those suffering from clinical depression. About 45% with a pro-inflammatory profile were identified in a study of both men and women from Georgia, USA (Raison et al., 2013) and fewer individuals (39%) with indications of an inflammatory phenotype were identified in a study from the UK (Lynall et al., 2020). Furthermore, we have previously shown that women with antenatal depression could display lower levels of inflammatory makers (mostly, but not only, anti-inflammatory markers) compared to non-depressed (Edvinsson et al., 2017). Taken together, the results point to major challenges when investigating associations between depressive symptoms and immune related markers in the perinatal period: Not only does the immune response change over pregnancy, but we expect that depressive symptoms are not associated with inflammatory alterations in all individuals and among those who do have alterations in immune function, changes might show as both increase and decrease in immune activation. Despite these challenges, this study adds information regarding potential differences in the immune response during the different phases of pregnancy in at least some women with mental distress.

### 4.1 Strengths and limitations

Some strengths of the current study are the population-based design, the longitudinal sampling, the availability and control for several potential confounders and the inclusion of non-pregnant controls for visualization purposes. The use of several trajectories of perinatal depression can also be considered novel in the field. However, this study has some limitations. Four interesting markers (IL-1beta, IL-6, IL-4 and IL-10) were excluded from further analyses due to levels under LOD for more than 25% of the samples, hence no IL-8/IL-10 ratio could be calculated (as in (Corwin et al., 2015)). Notably, the missing values for IL-6 were unevenly distributed between the different time-points, and only 1.8% were lower than the limit of detection at the delivery time-point. Due to the large number of overall missing values for IL-6, this marker was excluded from the statistical analyses.

Future studies validating the results are merited, as some of the subgroups’ sample sizes were small since not all women had donated samples at all time-points. This could have led to power issues. Variables of interest solely in distinct time-points, such as delivery mode or breastfeeding, could not be adjusted for in this type of statistical analysis. Further, due to the small sample size, it has not been feasible to conduct sub analyses including such variables, e.g. delivery mode, only impacting on the delivery time point and breastfeeding, for the postpartum time point; future studies with larger groups are encouraged. The Luminex panel, used for analyzing the cytokine levels, presents a broad dynamic range. However, comparison to classical ultra-sensitive immunoassays should be made with caution, as differences between methods have been observed (Martos-Moreno et al., 2010). Furthermore, a self-screening tool and not a clinical interview or diagnosis were used for assessment of depressive symptoms. While the subgrouping of women with perinatal depressive symptoms is a strength of this study, it might need to be further adjusted according to both symptom onset and which symptoms that are most dominant (Paul and Corwin, 2019; Putnam et al., 2017).

## 5 Conclusion

This study revealed the importance of timing of blood sampling peripartum, which should be taken into account in future studies that seek to examine inflammation-associated perinatal complications. Lower levels of VEGF-A in antepartum and postpartum depressed women throughout the perinatal period suggest possible immunological aberrations in these women. Further research is needed to determine mechanisms by which differences in adaptations of the immune response are associated to maternal depressive symptoms.

## Funding

Financial support for this study was provided by 10.13039/501100006285Magnus Bergvalls stiftelse (Dnr, 2017–02165), the 10.13039/100005930Swedish Research Foundation projects VR: 521-2013–2339/523-2014-2342, and the 10.13039/501100011898Marianne and Marcus Wallenberg Foundation (MMW2011.0115). The funding sources had no involvement in study design, in the collection, analysis and interpretation of data, in the writing of the report, or in the decision to submit the article for publication.

## Declaration of competing interest

The authors declare that they have no known competing financial interests or personal relationships that could have appeared to influence the work reported in this paper.
