# Supplementary material for: Longitudinal assessment of inflammatory markers in the peripartum period by depressive symptom trajectory groups
Source: Brain Behav Immun Health. 2022 May 2;22:100468. doi: 10.1016/j.bbih.2022.100468 (PMC9097612; doi:10.1016/j.bbih.2022.100468)
Supplement: Multimedia component 1 [file mmc1.docx]

# 8. Supplementary material

**Supplementary table 1.** Correlation coefficients of cytokines between time-points and between cytokines

|  | **Frak-talkine_w18** | **Frakt-alkine_w38** | **Frakt-alkine_delivery** | **Frakt-alkine_pp** | **TNF_w18** | **TNF_w38** | **TNF_delivery** | **TNF_pp** | **IL-8_w18** | **IL-8_w38** | **IL-8_delviery** | **IL-8_pp** | **IL-18_w18** | **IL-18_w38** | **IL-18_delivery** | **IL-18_pp** | **M-CSF_w18** | **M-CSF_w38** | **M-CSF_delivery** | **M-CSF_pp** | **VEGF-A_w18** | **VEGF-A_w38** | **VEGF-A_delivery** | **VEGF-A_pp** |
| --- | --- | --- | --- | --- | --- | --- | --- | --- | --- | --- | --- | --- | --- | --- | --- | --- | --- | --- | --- | --- | --- | --- | --- | --- |
| **Fraktalkine_w18** | 1,000 | **.520^**^** | **.669^**^** | **.509^**^** | 0,110 | 0,083 | 0,100 | 0,035 | -0,043 | **.358^*^** | -0,091 | 0,029 | 0,090 | 0,088 | 0,114 | 0,128 | 0,124 | 0,159 | 0,133 | **.241^*^** | -0,042 | -0,019 | 0,027 | -0,058 |
| **Fraktalkine_w38** |  | 1,000 | **.831^**^** | **.483^**^** | -0,259 | 0,148 | 0,119 | -0,242 | -0,154 | 0,188 | 0,221 | -0,209 | -0,131 | 0,043 | **.309^*^** | -0,087 | -0,297 | 0,143 | 0,168 | -0,258 | -0,088 | 0,085 | **.346^*^** | 0,062 |
| **Fraktalkine_delivery** |  |  | 1,000 | **.538^**^** | -0,060 | 0,028 | 0,147 | -0,075 | -0,025 | 0,196 | 0,107 | -0,053 | 0,100 | 0,126 | **.278^**^** | 0,169 | 0,005 | 0,114 | **.209^*^** | 0,083 | 0,078 | 0,097 | **.277^**^** | -0,008 |
| **Fraktalkine_pp** |  |  |  | 1,000 | 0,063 | -0,127 | 0,049 | 0,079 | 0,069 | 0,197 | 0,120 | 0,031 | 0,185 | 0,049 | 0,182 | **.214^*^** | -0,001 | -0,022 | 0,091 | **.205^*^** | -0,049 | **.310^*^** | 0,192 | 0,037 |
| **TNF_w18** |  |  |  |  | 1,000 | 0,298 | **.469^**^** | **.421^**^** | **.339^**^** | -0,010 | **.217^*^** | -0,024 | **.305^**^** | 0,099 | 0,032 | 0,107 | **.348^**^** | -0,163 | 0,005 | 0,117 | **.212^*^** | 0,112 | 0,023 | 0,067 |
| **TNF_w38** |  |  |  |  |  | 1,000 | **.512^**^** | **.377^**^** | 0,208 | **.481^**^** | 0,230 | 0,207 | -0,128 | -0,083 | 0,031 | -0,071 | -0,146 | **.303^*^** | 0,255 | 0,070 | 0,078 | 0,145 | 0,125 | 0,214 |
| **TNF_delivery** |  |  |  |  |  |  | 1,000 | **.332^**^** | 0,205 | 0,054 | **.493^**^** | **.203^*^** | 0,110 | 0,057 | **.216^*^** | 0,086 | **.238^*^** | **.289^*^** | **.395^**^** | 0,165 | 0,125 | -0,021 | 0,156 | 0,040 |
| **TNF_pp** |  |  |  |  |  |  |  | 1,000 | 0,035 | 0,250 | **.298^**^** | **.447^**^** | 0,207 | 0,029 | 0,121 | **.344^**^** | 0,064 | 0,184 | 0,191 | **.455^**^** | 0,113 | 0,135 | 0,125 | **.318^**^** |
| **IL-8_w18** |  |  |  |  |  |  |  |  | 1,000 | 0,186 | 0,110 | 0,021 | 0,092 | -0,175 | 0,005 | -0,152 | 0,166 | -0,050 | -0,031 | -0,184 | **.382^**^** | -0,021 | 0,073 | 0,002 |
| **IL-8_w38** |  |  |  |  |  |  |  |  |  | 1,000 | 0,213 | **.363^**^** | -0,196 | -0,114 | -0,085 | 0,076 | 0,079 | 0,067 | 0,024 | -0,081 | 0,126 | **.334^**^** | 0,184 | 0,199 |
| **IL-8_delviery** |  |  |  |  |  |  |  |  |  |  | 1,000 | 0,163 | 0,101 | -0,030 | 0,154 | 0,050 | 0,055 | **.311^*^** | **.341^**^** | 0,127 | 0,118 | 0,085 | **.211^*^** | 0,108 |
| **IL-8_pp** |  |  |  |  |  |  |  |  |  |  |  | 1,000 | 0,155 | 0,142 | 0,037 | **.298^**^** | -0,102 | -0,013 | -0,035 | **.265^**^** | 0,004 | 0,139 | -0,012 | **.343^**^** |
| **IL-18_w18** |  |  |  |  |  |  |  |  |  |  |  |  | 1,000 | **.625^**^** | **.597^**^** | **.554^**^** | **.285^**^** | -0,146 | -0,009 | 0,196 | 0,105 | -0,289 | 0,088 | 0,109 |
| **IL-18_w38** |  |  |  |  |  |  |  |  |  |  |  |  |  | 1,000 | **.725^**^** | **.678^**^** | 0,063 | 0,159 | -0,034 | 0,093 | 0,025 | 0,184 | 0,182 | **.299^*^** |
| **IL-18_delivery** |  |  |  |  |  |  |  |  |  |  |  |  |  |  | 1,000 | **.597^**^** | 0,074 | 0,118 | **.209^*^** | 0,163 | 0,117 | 0,097 | **.356^**^** | 0,132 |
| **IL-18_pp** |  |  |  |  |  |  |  |  |  |  |  |  |  |  |  | 1,000 | 0,067 | 0,164 | -0,040 | **.414^**^** | -0,068 | 0,225 | 0,097 | **.401^**^** |
| **M-CSF_w18** |  |  |  |  |  |  |  |  |  |  |  |  |  |  |  |  | 1,000 | **.427^**^** | **.511^**^** | **.441^**^** | 0,140 | -0,105 | 0,043 | -0,104 |
| **M-CSF_w38** |  |  |  |  |  |  |  |  |  |  |  |  |  |  |  |  |  | 1,000 | **.752^**^** | **.587^**^** | -0,136 | 0,094 | 0,116 | -0,016 |
| **M-CSF_delivery** |  |  |  |  |  |  |  |  |  |  |  |  |  |  |  |  |  |  | 1,000 | **.484^**^** | 0,058 | -0,100 | **.193^*^** | -0,088 |
| **M-CSF_pp** |  |  |  |  |  |  |  |  |  |  |  |  |  |  |  |  |  |  |  | 1,000 | -0,047 | 0,010 | 0,133 | **.276^**^** |
| **VEGF-A_w18** |  |  |  |  |  |  |  |  |  |  |  |  |  |  |  |  |  |  |  |  | 1,000 | 0,300 | **.271^*^** | **.297^**^** |
| **VEGF-A_w38** |  |  |  |  |  |  |  |  |  |  |  |  |  |  |  |  |  |  |  |  |  | 1,000 | 0,233 | **.444^**^** |
| **VEGF-A_delivery** |  |  |  |  |  |  |  |  |  |  |  |  |  |  |  |  |  |  |  |  |  |  | 1,000 | **.212^*^** |
| **VEGF-A_pp** |  |  |  |  |  |  |  |  |  |  |  |  |  |  |  |  |  |  |  |  |  |  |  | 1,000 |

**Supplementary table 2.** Pairwise comparisons of cytokine levels between time-point.
